# Prosocial modelling matters: the association between parent and faculty involvement in fighting COVID-19 with medical students’ career commitment

**DOI:** 10.1080/07853890.2022.2139410

**Published:** 2022-11-04

**Authors:** Dan Wang, Xiaoyang Ye, Hongbin Wu

**Affiliations:** a National Center for Health Professions Education Development, Peking University, Beijing, China; b Annenberg Institute for School Reform, Brown University, Providence, USA; c Institute of Medical Education/National Center for Health Professions Education Development, Peking University, Beijing, China

**Keywords:** Career choice, COVID-19, medical student, prosocial modelling, role model

## Abstract

**Background:**

Role models are essential in medical education, yet empirical research is relatively insufficient on the influence of prosocial modelling on medical students’ career commitment. The prosocial behaviour of medical staff involved in the fight against the novel coronavirus disease 2019 (COVID-19) at the beginning of 2020 presents an opportunity to fill the research gap. We explored and compared the different associations of the two most important role models for medical students – parents and faculty- with medical students’ career commitment.

**Methods:**

The cross-sectional study was conducted with 99,559 undergraduate students majoring in clinical medicine in mainland China. Questions were asked to collect information about participants in the battle against COVID-19, medical students’ determination to practice medicine after graduation, as well as students’ socio-demographic characteristics. Chi-square tests and hierarchical regressions were performed to examine the associations between parent and faculty involvement and students’ career commitment.

**Results:**

The results showed statistically significant associations between prosocial modelling during the COVID-19 pandemic in China and students’ intention to pursue medical careers. The association of faculty involvement (OR = 1.165, *p* < .001) with students’ career commitment was greater than that of parents (OR = 0.970, *p* > .05). For faculty involvement, the association was stronger among male students (OR = 1.323, *p <* .001) and students who were already determined to be doctors (OR = 1.219, *p* < .001) before the pandemic.

**Conclusions:**

Our study provides new evidence on the potential roles of parents and faculty in shaping medical students’ career commitment. Encouraging faculty to act as positive role models could help medical students increase their intention to become doctors.

**KEY MESSAGES**
- Prosocial modelling could enhance students’ intention to pursue medical careers.
- The association of prosocial behaviour of faculty is larger than that of parents on medical students.
- Those who have prior medical career commitment are much more likely to persist in the medical profession, and prosocial modelling of faculty is positively associated with their medical career commitment.

## 1. Introduction

The shortage of healthcare workers is a global problem. The World Health Organization (WHO) and the Association of American Medical Colleges (AAMC) have both published reports to emphasize the shortage of healthcare workers [1,2]. A high attrition rate among medical students, which leads to a significant loss of the accumulated human capital in medicine, expands the concern [3–5]. Thus, it is imperative to promote students’ willingness and commitment to stay in the medical profession. This paper investigates the role of prosocial modelling with this concern.

Prosocial behaviour, an action that benefits or promotes others [6], is a direct product of social learning [7]. Modelling plays an essential role in the form of prosocial behaviour [8]. Prosocial modelling is one of the major approaches to fostering prosocial behaviour, which could be a strategy to be used in educational institutions [7]. Highly prosocial medical workers have been found to have high levels of professional commitment [9]. In late January 2020, the novel coronavirus disease 2019 (COVID-19) was first reported in Wuhan, the capital of Hubei province in China. It has since spread rapidly across the world. As the medical resources in Hubei, the epicentre of COVID-19, were insufficient to deal with the outbreak, tens of thousands of medical staff were deployed from all over China to provide medical aid for the province, among whom more than 20,000 of those staff were from hospitals affiliated with medical colleges [10]. Numerous non-healthcare workers (particularly government officials and social workers) also actively participated in the fight against COVID-19. Their contributions are considered to be a form of prosocial behaviour [11,12].

This study presents the first empirical evidence of the association between this prosocial behaviour during the pandemic and medical students’ intention to pursue medical careers. We focus on the frontline involvement of the parent and faculty in fighting COVID-19 as a type of prosocial behaviour by using a nationally representative sample of undergraduate medical students in China (*N* = 99,559). We identified the heterogeneous associations between parent and faculty as prosocial modelling and students’ commitment to their careers in medicine.

Moreover, medical students vary in their individual characteristics. Research has shown that gender is a crucial factor affecting career choice [13–16]. Students from various learning phases interact differently with their faculty, and their relationship with their faculty impacts their career choice. Although the higher the grade level, the closer the contact with the faculty may be, some studies also demonstrated that medical students become more stressed as their grade level increases [17]. How students from different learning stages respond to prosocial modelling is still unknown. Furthermore, during the pandemic of COVID-19, the severity varied significantly across regions. Students’ psychological states and teachers’ participation in the different areas at the beginning of the epidemic also differed. Therefore, the study proposed three research questions: (1) Would the prosocial behaviour of role models during the COVID-19 outbreak be associated with medical students’ commitment to their careers? (2) Does the association of the parental modelling differ from that of the faculty modelling? (3) Does the observed association differ by students’ specific characteristics?

## 2. Institutional background and previous literature

### 2.1. Medical education in China

Medical education in China begins after senior high school. Students choose to enter university with a medical specialty after graduating from high school, and most students enter undergraduate at the age of 18. Many students choose medical majors without a clear understanding and long-term commitment to the profession; instead, they make the major choices due to many other factors, such as recommendations from parents, teachers, and friends. For example, the China Medical Student Survey shows that only 55% of medical students had the career aspiration of being a doctor in high school [4]. In addition, based on the national administrative data on the licensure examination (an examination that is required to obtain to practice medicine after graduating), approximately 20% of medical students do not take the examination each year when they are eligible to do so [5]. Related studies have also reported high attrition rates among Chinese medical students [3,18,19]. Although some Chinese medical education experts have pointed out that the study’s data were misquoted [20], the high attrition rate of Chinese medical students is indisputable.

### 2.2. Parent and faculty role models in medical education

Parents, who are typically role models for their children, have a meaningful influence on their offspring [21]. A study showed that teenagers’ plans could be influenced by their parents as role models [22]. Another survey demonstrated that nearly half of the surveyed medical school applicants had family members with medical backgrounds [23]. For many children, their parents act as the initial and most significant role models to help shape their attitudes and behaviours. However, they may seek a variety of role models after entering school, at which stage the teaching faculty can become influential. Faculty role models are important in medical education. Their significance to the professional development of medical students has been confirmed by medical educators worldwide [24–27]. Medical students may seek to emulate role models’ passion for their careers [28]. Many studies have agreed on the influence of parents and faculty on the intention of medical students to become doctors, but empirical evidence of the different effects of parents and faculty is lacking.

Previous studies have, however, indicated that these influences might differ. Parents of pre-school children and school teachers were assessed as role models regarding the children’s healthy lifestyles, and the results showed that the parents but not school teachers had a more significant impact [29]. Studies of adolescent students have demonstrated that they are more likely to leave their parents and seek help from teachers [30]. But limited studies have compared the effects of parental and faculty behaviours on emerging adults like medical students. Family influence may be a long-term process, and it is hard to distinguish the parents’ effect alone. The involvement of medical staff in fighting COVID-19 has provided an opportunity to study the impact of the same behaviour of parents and faculty on medical students simultaneously.

## 3. Methods

### 3.1. Participants and survey

The cross-sectional study was conducted between 21 February and 14 March 2020, among all undergraduate clinical medicine students at 90 institutions in mainland China. Specifically, the national scale, multi-site survey was performed in China with the support of the National Centre for Health Professionals Education Development. After a sampling process stratified by geographical location, type of institution, and reputation of the medical schools, 90 randomly selected medical schools from stand-alone medical colleges or comprehensive universities were invited to participate in the survey. According to the sample size calculation rules [31], the sample size to meet the requirements of our study should be greater than 2,844. Between 21 February and 14 March 2020, when the epidemic in Hubei Province was basically under control, we distributed the questionnaire via the professional version of the online survey platform WJX (https://www.wjx.cn/). Each participating medical school sent a formal survey invitation to all undergraduate clinical medicine students (*N* = 225,329). Participation was completely voluntary and anonymous. An online disclosure statement and a consent agreement were provided to them before the survey was started. The inclusion criteria for respondents were (a) from our 90-sample schools, (b) undergraduate students, (c) clinical medicine specialty, (d) willing to provide online consent. This survey received a total of 118,030 responses (response rate = 52.38%) [32]. After returning all questionnaires, we cleaned the data according to the exclusion criteria (a) the answer was illogical, (b) students completed the survey in fewer than 5 min as it was too short to complete the questionnaire properly. Finally, we obtained 99,559 valid observations (effective rate = 84.35%). The survey was unique to each participant and could not be shared or completed more than once.

### 3.2. Dependent variable

The survey item ‘*The pandemic has made me more committed to my future career as a doctor?*’ was used to assess whether the pandemic increased the intention of the students to pursue medical careers. Using a 5-point Likert scale, the study identified those who reported ‘strongly agree’ (5) or ‘agree’ (4) as having an increased intention to become doctors.

### 3.3. Independent variables

We used two questions to measure parent and faculty involvement in the COVID-19 outbreak. The question ‘*Does either of your parents serve on the frontline against COVID-19?*’ was used to measure parental participation, with three possible responses: ‘both parents’, ‘one parent’, or ‘neither parent’. We combined options 1 and 2 to indicate parental involvement (the results were similar when we examined the two items separately). The question ‘*Have your faculty members gone to Hubei province to take part in the battle against COVID-19?*’ was used to measure faculty involvement, and students could enter either a ‘yes’ or ‘no’ response. Our study compares the impact of the anti-pandemic behaviour of parents and faculty involved in the most severe areas of the pandemic on medical students. To exclude cases where parents were medical workers but failed to participate in the fight against COVID-19 and cases where parents were not medical workers but participated in the fight (volunteer or manager on the frontline). We also asked ‘*Are either of your parents doctors?*’ to examine whether there are parents in the medical profession. The options were ‘both parents’, ‘one parent’, or ‘no’. We combined 1 and 2 to indicate that the respondents had one or two doctors as parents. We then divided the medical students into four groups. Those in group 1 knew no one who had gone to Hubei province to assist in the battle against COVID-19; group 2 only knew faculty members who had participated; group 3 consisted of those whose parents had gone to Hubei; those in group 4 both had parents and knew faculty who had participated in the battle against COVID-19. Each group was then divided into two types according to whether their parents were doctors.

### 3.4. Control variables

We also considered other control variables, including gender, self-reported academic performance (top 25%, 25%–50%, 50%-75%, bottom 25%), learning phase, home location (urban or rural), the region of the medical schools, and whether the students had planned to become doctors before the pandemic. Although academic performance has limitations as a self-reported variable, the meaning it specifies can help us understand more deeply the factors that influence the dependent variable. The learning phases were divided into four phases according to the clinical medical training phases [33]. The regions in which the medical schools were located were separated into three according to the classification of the National Health Commission of the People’s Republic of China [34]. Considering the high proportion of Chinese medical students who chose not to practice medicine, ‘planned to become doctors before the pandemic’ can be used to measure medical students’ commitment to the profession before the pandemic. This pre-pandemic baseline measure helps address the potential omitted variable bias issues by controlling for other unobservable covariates correlated with the outcome. Therefore, the study can measure the changes in career commitment due to parent and faculty involvement in fighting COVID-19 during the pandemic.

### 3.5. Statistical analysis

The study first established summary descriptive statistics of the variables. Chi-square tests were used to examine the relationships between types of role models and the intention of medical students to become doctors. Logistic models in which hierarchical regression were applied to estimate the effects. Parental involvement and faculty involvement in the battle against COVID-19 were included in the model as the first step. The students’ willingness to become doctors before the pandemic was included as the second step. The third step includes characteristics of the students, such as their gender, home location, academic performance, college region, and learning phase. Finally, to explore the heterogeneous effects, we chose the four variables: prior career commitment, gender, learning phase, and region. Using these moderating variables, we estimated the effects of parental and faculty involvement separately. The significance level was set to 0.05. All analyses were performed using Stata SE15.

## 4. Results

In this section, the study explored the role of modelling on medical students’ career commitment. It also compared the different modelling between the parent and faculty. Lastly, the heterogeneity of these two role models across different groups of medical students was analysed.

### 4.1. Demographic characteristics

In total, 118,030 medical students responded to the survey (with a response rate of 52.38%), and the final sample consisted of 99,559 valid responses. The characteristics of the study participants are summarized in Table 1. This study included 60,815 (61.08%) female and 38,744 (38.92%) male students. It should be noted that more students attended the eastern and central than the western schools, which conforms to the general proportions of students at Chinese medical schools. Before the COVID-19 outbreak, 85,922 students (86.30%) wanted to become doctors after graduation, and 13,637 (13.70%) did not.

**Table 1.** Summary statistics for medical students in the study (*n* = 99,559).

Variables	Participants, *N* (%)	Nationally, %
Gender		
Female	60,815 (61.08)	59.36^a^
Male	38,744 (38.92)	40.64^a^
Academic performance		
Bottom 25%	6,320 (6.35)	
50%–75%	21,112 (21.20)	
25%–50%	35,687 (35.85)	
Top 25%	36,440 (36.60)	
Region		
Eastern	38,134 (38.30)	31.20^a^
Central	41,341 (41.53)	32.94^a^
Western	20,084 (20.17)	35.86^a^
Area (home location)		
Rural	41,538 (41.72)	46.02^a^
Urban	58,021 (58.28)	53.98^a^
Learning phase		
General education	14,774 (14.84)	
Basic medical education	43,026 (43.22)	
Clinical medical education	30,869 (31.00)	
Clerkship rotation	10,890 (10.94)	
Willingness to become a doctor before the pandemic.		
No	13,637 (13.70)	
Yes	85,922 (86.30)	

^a^Data Source: 2020 China Medical Student Survey (CMSS), conducted by the National Centre for Health Professionals Education Development (NCHPED), authorized by the Ministry of Education (MOE) and National Health Commission (NHC) [4].

### 4.2. Chi-square test results

To understand the relationship between prosocial modelling and the increased intention of medical students to become doctors, the study compared the parent and faculty involvement groups, as shown in Table 2. 66.47% (*N* = 66,180) students had an acquaintance (either a parent or a faculty member) who had assisted in the fight against the COVID-19 outbreak. Of these, 47,940 (72.44%) expressed a greater intention to pursue a medical career after the pandemic. Among students without such acquaintances, 22,930 (68.70%) held the same intention. The chi-square test results revealed a statistically significant difference in the intention to pursue a medical career between the two groups (*p* < .001).

**Table 2.** The relationship between role models and medical students’ intention to become doctors.

	The COVID-19 pandemic has enhanced my commitment to be a doctor		
	No *n* = 28,689(%)	Yes *n* = 70,870(%)	Chi-square
	(28.82)	(71.18)	
Have an acquaintance who has participated in anti-pandemic activities			151.5***
No	10,449 (31.30)	22,930 (68.70)	
Yes	18,240 (27.56)	47,940 (72.44)	
Parents are doctors			30.2***
Neither involvement	814 (33.18)	1,639 (66.82)	
Faculty involvement	1,103 (27.71)	2,877 (72.29)	
Parents involvement	298 (30.28)	686 (69.72)	
Both faculty and parents’ involvement	752 (27.00)	2,033 (73.00)	
Parents are not doctors			137.6***
Neither involvement	9,635 (31.16)	21,291 (68.84)	
Faculty involvement	15,281 (27.61)	40,058 (72.39)	
Parents involvement	233 (29.05)	569 (70.95)	
Both faculty and parents involvement	573 (25.02)	1,717 (74.98)	

***p < .001.

To further examine the different effects of parents and faculty members as prosocial role models, we grouped the medical students according to their acquaintances and parents’ occupations and then conducted the chi-square tests. The results indicated a statistically significant difference between these groups, whether parents are doctors or not (*p* < .001). The details are given in Table 2.

### 4.3. Hierarchical regression results

A hierarchical regression model was applied to identify the degree of correlation between the independent and dependent variables while avoiding mutual interference between the variables. This hierarchical regression analysis involved four steps: the first included only parental and faculty involvement, the second added willingness to become a doctor before the pandemic, the third added sociodemographic indicators such as gender, region and learning phase, and the fourth step added academic performance. Table 3 provides the results of the hierarchical regression. According to step 4, We found that the increased intention to pursue a medical career was lower for students whose parents were doctors compared with the base group (OR = 0.864, *p* < .01). The participation of faculty significantly associated with increased commitment of medical students to pursuing future medical practice (Faculty and doctor parents: OR = 1.165, *p* < .001; Faculty and non-doctor parents: OR = 1.263, *p* < .001), while parent participation played no significant role (Parents and doctor parents: OR = 0.970, *p* = .687; parents and non-doctor parents: OR = 1.093, *p* = .286). After controlling for other factors, when the students’ parents were doctors, and the students were acquainted with faculty who had participated in the fight against COVID-19, their intention to become doctors was higher than that of the students in the control group (whose parents were not doctors and who lacked acquaintances). However, no significant difference was found in the intentions of the students whose parents had participated in the pandemic compared with the control group, whether their parents were doctors or not. In the last column of Table 3, we also give the 95% confidence interval values for step 4.

**Table 3.** Hierarchical regression results (*n* = 99,559).

Variables	Step 1	Step 2	Step 3	Step 4	95% Confidence Interval, %
Participation (base: neither & parents are not doctors)					
Neither involvement & Parents doctors	0.911*	0.888*	0.868**	0.864**	0.788–0.948
Faculty involvement & Parents doctors	1.186***	1.160***	1.177***	1.165***	1.078–1.259
Faculty involvement & Parents not doctors	1.180***	1.119**	1.245***	1.236***	1.196–1.277
Parents involvement & Parents doctors	1.042	0.998	0.984	0.97	0.839–1.122
Parents involvement & Parents not doctors	1.105	1.096	1.084	1.093	0.929–1.286
Both involvement & Parents doctors	1.223***	1.147**	1.201***	1.180***	1.077–1.294
Both involvement & Parents not doctors	1.356***	1.324***	1.402***	1.388***	1.252–1.539
Key independent variable					
Wanted to become a doctor before the pandemic		5.465***	5.509***	5.397***	5.192–5.609
Demographics					
Male			1.029	1.060***	1.029–1.092
Urban			0.993	0.989	0.960–1.019
Region (base: eastern)					
Central			1.183***	1.171***	1.134–1.210
Western			0.971	0.967	0.930–1.006
Learning phase (base: general education)					
Basic medical education			0.848***	0.852***	0.814–0.892
Clinical medical education			0.600***	0.622***	0.593–0.652
Clerkship rotation			0.503***	0.528***	0.499–0.560
Academic performance (base: bottom 25%)					
50%-75%				1.191***	1.119–1.267
25%-50%				1.341***	1.263–1.423
Top 25%				1.535***	1.445–1.630

‘Neither’ means neither parents nor faculty. ‘&’ indicates that two conditions are met at the same time. For example, ‘Faculty involvement & parents are doctors’ means that faculty participated in the pandemic and students’ parents are also doctors. Robust standard errors are clustered at the college level. ***p < .001, **p < .01, *p < .05.

The students’ intention to become doctors before the pandemic significantly affected their subsequent intentions to become doctors. The students who wanted to become doctors before the pandemic were five times more committed than those who did not (wanted to become a doctor before the pandemic: OR = 5.397, *p* < .001). Also, the results revealed that the male students had a greater intention to practice medicine than the female students (Male: OR = 1.060, *p* < .001). Those with better grades were also more inclined to become doctors. The increased intention to pursue a medical career was higher for those from the central region than those from the eastern region, followed by those from the western region, as the higher the learning stage, the lower the increased intention.

### 4.4. Heterogeneity

We applied logistic models with hierarchical regression with four variables: prior career commitment, gender, learning phase, and college region. Each logistic model was used to explore the association between parents’ and faculty’s participation in the fight against COVID-19 with medical students’ intention to become doctors. The results are shown in Table 4. Having a parent as a doctor turned out to be a negative impact factor. To ease the discussions, we only report the odd ratio values of the participation of faculty and the parent when the parents were doctors.

**Table 4.** Comparison of the faculty participation and parent participation when parents are doctors (*n* = 4,964).

Variables	Faculty involvement	Parents involvement
Prior career commitment		
Did not want to become a doctor	0.894	0.733
Wanted to become a doctor	1.219***	1.015
Gender		
Female	1.052	0.991
Male	1.323***	0.955
Learning phase		
General education	1.199	1.304
Basic medical education	1.108	0.880
Clinical medical education	1.132	0.896
Clerkship rotation	1.333	1.430
Region		
Eastern	1.183*	1.015
Central	1.133*	0.897
Western	1.213	1.049

Each row represents a logistic model. We only show the OR values of the participation of parents and faculty when the medical students’ parents were doctors. The logistic model: medical students’ intention = log (xi | *V* = 1), xi includes group, whether students wanted to be doctors before the pandemic, gender, academic performance, learning phase, home location, and region of college. V is the value of the variables. For example, if v = female, OR (Faculty & parents doctors) = 1.052, OR (Parents & parents doctors) = 0.991. Robust standard errors are clustered at the college level.

***p < .001, **p < .01, *p < .05.

For those students who had prior career commitment, faculty participation was significantly and positively associated with medical career commitment (OR = 1.219, *p <* .001). In contrast, the association of parents’ participation with medical career commitment was not significant (OR = 1.015, *p* = .858). For male students, the participation of faculty was related to the medical career commitment (Male & Faculty: OR = 1.323, *p < .001*), but the result of the participation of parent was not (Male & Parent: OR = 0.955, *p =* .625). For those students who have entered the rotation period of clinical practice, faculty participation was significantly associated with the medical career commitment at the 10% level (Clerkship rotation & Faculty: OR = 1.333, *p =* .061), while the participation of parents was not. The outcomes concerning regions were of interest. For students studying in the eastern and central regions, faculty participation showed significant association with a higher intention to pursue medicine as a career. In contrast, the participation of parents did not (Eastern & Faculty: OR = 1.183, *p <* .01; Central & Faculty: OR = 1.133, *p <* .05). For students studying in the western regions, neither faculty nor parent participation significantly impacts medical students’ career commitment.

## 5. Discussion

This study analyzed a large-scale, nationally representative sample of 99,559 students from 90 medical schools in China. We illustrated the association between the prosocial behaviour of role models with medical students’ intention to pursue a medical career. We found that the faculty’s prosocial behaviour significantly affected medical students, while parents did not. This study contributes to understanding the effects on medical students of exposure to role models’ frontline involvement in the fight against COVID-19.

Existing studies have shown that prosocial modelling promotes prosocial behaviour. For instance, students who watched a cartoon about helping puppies were more likely to help others than those who had not watched the cartoon [35]. Another study revealed that after observing an example of active blood donation, the probability that college students would donate blood was higher than that of students without the blood donation model [36]. Our study assumes that the doctor can offer a role model to medical students and confirms the positive effect that the prosocial behaviour of role models participating in the battle against the pandemic can have.

The results indicated that during the COVID-19 pandemic, prosocial modelling has been associated with medical students’ intention to pursue a medical career and that the effect of faculty was significant while parents did not. Studies of adolescent students have demonstrated that they are more likely to leave their parents and seek help from teachers [30]. From the perspective of the theory of the CoP, medical students are in a shared community of learning/practice with their faculty. They share the same ideas, and their occupational expectations are influenced most by other community members. Medical students join this community as novices and obtain knowledge and skills, along with the identity conferred by their community membership [37]. They hold the same goal of preventing and treating human diseases [38]. Medical students learn from faculty members what it is to be a doctor [39], and they may retain the faculty’s behaviour, which then affects their motivations and occupational expectations. The role of parents is perhaps reflected more in the choice of medical major [23]. Students are familiar with their parents but not necessarily with the faculty. Thus, the influence of faculty could have been underestimated. This again highlights the power wielded by faculty as role models [40,41], which is greater than the influence of family at this stage of learning. Doctors should be aware of the effect of role models and that by being good role models, they can attempt to avoid adverse impacts on medical students.

The willingness to become a doctor before the pandemic is significantly associated with medical students’ commitment. Compared with those who did not, students who wanted to become doctors before the pandemic demonstrated a more tremendous increase in the intention to pursue a medical career. Factors that can influence medical students’ career choices include the potentially high income, their status as perceived by their colleagues, and opportunities to benefit their families [42,43]. Altruistic and scientific reasons are the main motivations for medical students’ career choices [44], but the primary motivation can differ between countries and depend on the level of income [45].

We found that during the COVID-19 pandemic, male students were more affected by role models than female students. A survey conducted in Japan found that women prefer disciplines such as paediatrics, gynaecology, obstetrics, and psychology, while men prefer orthopaedics and operating [46]. Many studies have proven that medical students of different gender have different motivations [47]. A controllable lifestyle has been found to be an important factor in women’s choices [16], and women have been shown to attach more importance to family and lifestyle than men [48]. The social definition of the male role is often more independent, with a stronger sense of social responsibility. Masculine traits such as strength and tenacity can also be viewed as attracting especial respect [49]. Thus, men may be more encouraged than women by others’ involvement in the fight against the pandemic and become more determined to pursue medical careers.

Those majoring in clinical medicine in China typically go through four phases. The first is general education, in which the main courses are chemistry, mathematics, sociology, biology, computer science, and English. The second phase is basic medical education, which includes anatomy, histology and embryology, biochemistry, and physiology. The third phase is clinical medical education, which includes introductory clinical courses such as internal medicine, surgery, obstetrics, gynaecology, paediatrics, and diagnostics. The fourth phase is clerkship rotation, in which students’ study with the clinical faculty of various departments in a hospital [33]. Some studies have shown that before entering medical college, the mental health levels of medical students are the same or higher than those of their peers [50,51], but after starting medical school their mental health can deteriorate [52–54]. Relevant research has suggested that newly enrolled medical students have excessive expectations for professional happiness, and could be easily affected by interventions [55]. Senior students are particularly likely to suffer from burnout [53,56,57], which is related to the level of stress they experience. Good support can, however, reduce the possibility of burnout [17], and good communication with faculty can have a positive impact on students’ learning perceptions [58]. Our research demonstrated that faculty might positively affect medical students’ intention to pursue a medical career when in the clerkship rotation phase, while parents do not. The rotation phase is when medical students have the closest contact with faculty, and it is also the critical phase in which medical students imitate their role models. The experiences of medical students in the clinical phase thus significantly affect their choices. The study confirms the significant role of faculty’s positive guidance. Although the pandemic affects the arrangement of rotation, students of this learning phase may be more sensitive than others.

We found a statistically significant association between the region and the increased intention to pursue medicine as a career. The central region, as the epicentre of the pandemic, was most sensitive to it, and thus the anti-pandemic behaviour of medical staff in the central region was more extensive. Thus, the prosocial behaviour of role models was more apparent. According to figures released by the National Health Commission of the PRC, the eastern region sent the most medical personnel of all regions of China [59]. Half of the medical staff participating in anti-pandemic work were from affiliated hospitals of the university, and thus typically taught medical students as faculty. The more local medical faculty involved in the fight against COVID-19, the greater students intended to become doctors. While the western region, for reasons of its economic development, sent relatively few staff to fight COVID-19 and was also relatively weakly affected by the pandemic. There was, therefore, no significant impact. In this study, we do not report the effect of the number of people in the fight on medical students’ career commitment, and we will include the number of people in the fight as an indicator of the intensity of prosocial behaviour discussed in our future study. For the eastern and central regions, faculty played a significant role in increasing the students’ intention to practice medicine, while the parent group did not. It indicates that the prosocial behaviour of faculty plays a greater role than that of parents in medical students’ intention to pursue a medical career in these regions, which is also consistent with our hypothesis.

The study has several limitations. First, we only measured the influence of parents in general, without distinguishing between the influences of fathers and mothers on their offspring. Some studies have reported that the influences of fathers and mothers on children differ [60]. Second, this study was conducted during the pandemic, so we did not consider the effects of the key variables on medical students after the pandemic. It is unclear how long the effect may last. Finally, this survey was self-reported, and thus may have included reporting and selection bias. We intend to address these limitations in the future.

## 6. Conclusions

The study analyzed completed questionnaires from 99,559 medical students from 90 medical schools in China. The findings showed that during the COVID-19 pandemic, the prosocial behaviour of role models had a positive association with medical students’ intention to pursue a medical career, which provides an opportunity to increase medical students’ willingness to pursue a career in medicine. The study also demonstrated that faculty role models had a more significant effect than parental role models, which means that faculty positive role models could have an even greater impact on medical students than contemporaneous parents. In addition, the effect varied among different student groups. Those who have prior medical career commitment are much more likely to persist in the medical profession and the prosocial modelling of faculty is associated with their medical career commitment. Male students were more affected by role models than female students. It suggests that encouraging faculty to act as positive role models could help medical students increase their career commitment. Furthermore, faculty should recognize the power of prosocial behaviour and demonstrate more positive role modelling in their daily teaching as well as medical activities, given that their words and actions have a crucial role for students in the profession. They should also be aware that there is a need to understand the differences in students and provide more targeted strategies to support their career advancement.

## Data Availability

The data used in this study are not publicly available due to confidentiality reasons. Data supporting the results of this study can be obtained from the author, Prof. Hongbin Wu.

## Additional information

Our paper has been pre-printed already. https://assets.researchsquare.com/files/rs-293789/v1/f2aea680-09de-4e1b-9db1-efee30ab2aa0.pdf?c=1637817259. DOI: https://doi.org/10.21203/rs.3.rs-293789/v1
